# Modulation of Gene Expression by 3-Iodothyronamine: Genetic Evidence for a Lipolytic Pattern

**DOI:** 10.1371/journal.pone.0106923

**Published:** 2014-11-07

**Authors:** Veronica Mariotti, Erika Melissari, Caterina Iofrida, Marco Righi, Manuela Di Russo, Riccardo Donzelli, Alessandro Saba, Sabina Frascarelli, Grazia Chiellini, Riccardo Zucchi, Silvia Pellegrini

**Affiliations:** Department of Surgical, Medical, Molecular Pathology and of Critical Care, University of Pisa, Pisa, Italy; University of Salento, Italy

## Abstract

3-Iodothyronamine (T_1_AM) is an endogenous biogenic amine, structurally related to thyroid hormone, which is regarded as a novel chemical messenger. The molecular mechanisms underlying T_1_AM effects are not known, but it is possible to envisage changes in gene expression, since delayed and long-lasting phenotypic effects have been reported, particularly with regard to the modulation of lipid metabolism and body weight. To test this hypothesis we analysed gene expression profiles in adipose tissue and liver of eight rats chronically treated with T_1_AM (10 mg/Kg twice a day for five days) as compared with eight untreated rats. *In vivo* T_1_AM administration produced significant transcriptional effects, since 378 genes were differentially expressed in adipose tissue, and 114 in liver. The reported changes in gene expression are expected to stimulate lipolysis and beta-oxidation, while inhibiting adipogenesis. T_1_AM also influenced the expression of several genes linked to lipoprotein metabolism suggesting that it may play an important role in the regulation of cholesterol homeostasis. No effect on the expression of genes linked to toxicity was observed. The assay of tissue T_1_AM showed that in treated animals its endogenous concentration increased by about one order of magnitude, without significant changes in tissue thyroid hormone concentration. Therefore, the effects that we observed might have physiological or pathophysiological importance. Our results provide the basis for the reported effectiveness of T_1_AM as a lipolytic agent and gain importance in view of a possible clinical use of T_1_AM in obesity and/or dyslipidaemia.

## Introduction

Thyroid hormones (THs) control adipose tissue development and metabolism [1]. They regulate adipocyte proliferation and differentiation [2], [3] and, as they cause weight loss by increasing the metabolic rate, may be indicated for obesity treatment [4]. Their use, however, is limited because they produce thyrotoxic effects including cardiotoxic effects like tachycardia and arrhythmia [4]. The identification of TH analogs that retain anti-obesity efficacy with a few undesirable side effects is therefore an important research goal.

Some TH derivatives have been recently shown to possess the same beneficial metabolic effects as THs without negative effects. In particular, the biogenic amine 3-Iodothyronamine (T_1_AM) may affect carbohydrate and lipid metabolism at doses that do not appear to produce undesirable side effects [5], [6].

Scanlan and colleagues demonstrated that T_1_AM is an endogenous component, which interacts with specific receptors (different from the nuclear thyroid hormone receptors) and produces significant functional effects, suggesting that it should be regarded as a novel chemical messenger [7]. By using an assay based on high performance liquid chromatography coupled to tandem mass spectrometry (HPLC-MS/MS) T_1_AM has been detected in rat serum and tissues as well as in human and Djungarian hamster blood [6], [8], [9]. The quantitative analysis of its physiological concentration showed that T_1_AM content is higher in organs than in blood, suggesting that some tissues are able to accumulate it [8].

The pathway of endogenous T_1_AM biosynthesis is still unknown. T_1_AM has been suggested to derive from THs through decarboxylation and deiodination [7], but the iodothyronine-decarboxylating enzyme has not yet been identified [10].

The physiological role of T_1_AM is still under investigation and different effects have been observed after T_1_AM administration. In rodents, intraperitoneal injections of T_1_AM induce bradycardia, hypothermia, hyperglycemia, decrease of metabolic rate (*V*O_2_), reduction of respiratory quotient (RQ), ketonuria and loss of fat mass [6], [7], [11]. The RQ decrease indicates that the utilisation of carbohydrates is suppressed in response to T_1_AM and that the energy requirements are covered by lipid consumption, and this conclusion was recently confirmed by metabolomics analysis and evaluation of carbon isotopic ratio [12]. Notably, at the doses used in the latter study, no effect on thermogenesis or cardiac function was detected.

T_1_AM may also produce effects on the central nervous system. There is evidence of a biphasic effect on food intake [13], and the metabolic effects described above might be mediated at least in part by changes in insulin and/or glucagone secretion which have been observed after i.c.v. administration [14], [15]. In addition, it has been recently suggested that T_1_AM may have pro-learning effects [16].

The molecular targets of T_1_AM are currently unknown. T_1_AM has been found to regulate the cAMP synthesis through the interaction with the G protein-coupled trace amine-associated receptor 1 (TAAR1) and possibly with other receptors of this class [7], [11], [17]. Snead and colleagues reported a regulation of membrane transporters like vesicular monoamine transporter (VMAT2) by T_1_AM, suggesting a neuromodulatory role for T_1_AM [18]. Interaction with α_2A_ adrenergic receptor (Adra_2A_) has been speculated to occur in pancreatic beta-cells [11].

It is still unknown whether T_1_AM has any effect on gene expression. Therefore, the aim of our study was to provide a comprehensive insight into T_1_AM transcriptional activity, by using microarray technology in rats chronically treated with T_1_AM, compared to untreated rats. Since the effects of T_1_AM on fatty acid metabolism appear to outlast all the other effects (bradycardia, hyperglycemia, hypothermia and hypometabolism) we choose to investigate gene expression in liver and adipose tissue, and discussed our results with special regard to their potential implications on lipid metabolism.

## Materials and Methods

### Animals and T_1_AM treatment

The animals used in this study were male Wistar rats. Prior to any experimental manipulation the rats were acclimatized for one week in the animal house facility of our Department.

The project was approved by the Animal Care and Use committee of the University of Pisa. Eight rats of 100–125 g body weight were treated with T_1_AM by intraperitoneal injection of 10 mg/Kg twice a day for five days. Eight control rats were treated with T_1_AM free-intraperitoneal injections under parallel housing conditions. The rats were then sacrificed by guillotine and the subcutaneous adipose tissue and liver were immediately removed, flash-frozen and stored at −80°C until their use.

T_1_AM was kindly provided by Prof. Thomas Scanlan, Oregon Health & Science University.

### Assay of T_1_AM

T_1_AM was assayed in samples of liver and adipose tissue by high performance liquid chromatography (HPLC) coupled to tandem mass spectrometry (MS-MS). Liver samples (50–200 mg) were homogenized on ice in 1.5 ml of phosphate buffer (154 mM NaCl, 6.7 mM NaH_2_PO_4_, pH 7.4) by 15+15 passes in a Potter-Elvejheim homogenizer. The homogenate was centrifuged for 10 min at 18620×g, the pellet was discarded and the supernatant was placed in a 15 ml centrifuge tube. After vortexing, 60 mg of NaCl was added; the mixture was equilibrated at room temperature for one hour and then deproteinized with 2 ml acetone in an ice bath for 30 min. After centrifugation at 720×g for 15 min the supernatant was evaporated to 1 ml using a Concentrator Plus (Eppendorf, Hamburg, Germany) kept at 30°C. Subsequent steps included solid phase extraction, HPLC separation and MS-MS assay, which were performed as described previously [8].

Adipose tissue (100–250 mg) was extracted for 30 min in 1 ml of acetonitrile and 0.1 M HCl (85∶15, v/v), in an ultrasound bath (LBS1 3Lt, Falc Instruments, Treviglio, Italy). The material was diluted to 2 ml with acetonitrile and homogenized by 12+12 passes in a Potter-Elvejheim homogenizer. The homogenate was further sonicated for 10 min, vortexed for 1 min and centrifuged at 720×g for 15 min. The supernatant was subjected for three times to liquid/liquid extraction with 1 ml hexane: the upper phase (hexane) was discarded and the lower phase (acetonitrile) was eventually dried under a gentle stream of nitrogen. The dried samples were reconstituted with 0.1 M HCl/methanol (50∶50, v/v) and subjected to HPLC separation and MS-MS assay, as described previously [8].

### Isolation, amplification and labelling of RNA

Total RNA was isolated from adipose tissue and liver by the RNeasy Lipid Tissue Mini kit (Qiagen, Valencia, CA, USA) and the RNeasy Microarray Tissue Mini kit (Qiagen, Valencia, CA, USA), respectively.

Residual DNA was eliminated by on-column DNase digestion using the RNase-Free DNAase Set (Qiagen, Valencia, CA, USA).

The quantity and purity of total RNA were measured by 260 nm UV absorption and by 260/280 ratio, respectively, using a NanoDrop ND-1000 Spectrophotometer (NanoDrop Technologies, Wilmington, DE, USA). All RNAs displayed a 260/280 optical density ratio ≥1.9.

RNA integrity was checked with the Agilent 2100 Bioanalyzer (Agilent Technologies, Palo Alto, CA, USA) using the Agilent RNA 6000 Nano kit (Agilent Technologies, Palo Alto, CA, USA). All RNAs displayed a RNA Integrity Number (RIN) ≥8.

One microgram of total RNA from treated and control animals was amplified and labelled with Cyanine 5 (Cy5) or Cyanine 3 (Cy3) dyes (Agilent Technologies, PaloAlto, CA, USA) by the Quick-Amp Labeling kit (Agilent Technologies, Palo Alto, CA, USA). In order to monitor the experiments from sample amplification and labelling to microarray hybridization a RNA Spike-In (Agilent Technologies, PaloAlto, CA, USA) was added to each RNA sample.

The Cy3 and Cy5 dye incorporation rates were measured by UV absorption at 555 nm and 647 nm, respectively. Both fluorophores showed a comparable incorporation efficiency ranging between 11 and 15 pmol of dye per µg of amplified RNA.

### Microarray hybridization

The hybridization mixture containing 825 ng of Cy3-labelled amplified RNA (corresponding to 9–10 pmol of Cy3 dye), 825 ng of Cy5-labelled amplified RNA (corresponding to 11–12 pmol of Cy5 dye), 11µl of 10X Blocking Agent, 2.2 µl of 25X fragmentation buffer and 55 µl of 2X GE hybridization buffer (the last three from the Gene Expression hybridisation kit plus, Agilent Technologies, Palo Alto, CA, USA) was hybridized to Whole Rat Genome Oligo Microarrays 4x44K G4131F (Agilent Technologies, Palo Alto, CA, USA). Each slide contains 4 arrays with 44,000 60-mer oligonucleotide probes representing 41,012 unique probes.

Array hybridisation was performed at 65°C in the Agilent oven G2545A (Agilent Technologies, Palo Alto, CA, USA) for 17 h under constant rotation. After hybridisation, the arrays were washed following the Quick Amp Labeling protocol (Agilent Technologies, Palo Alto, CA, USA). To prevent the ozone-mediated fluorescent signal degradation, the arrays were immersed in Acetonitrile solution (Sigma-Aldrich, St. Louis, MO, USA) for 10 sec and successively in Stabilization and Drying solution (Agilent Technologies, Palo Alto, CA, USA) for 30 sec. These last two washes were performed at room temperature.

### Microarray experimental design

**Figure 1:**
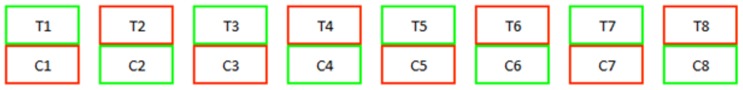
Balanced Block experimental design.

A balanced block design was applied separately for adipose and hepatic tissue samples: on each array, two differently labelled samples from the treated and the control groups were hybridized, for a total of eight arrays (figure 1).

### Microarray data acquisition and analysis

Microarray images were acquired by the Agilent scanner G2565BA (Agilent Technologies, Palo Alto, CA, USA) at 5 µm resolution and intensity raw data were extracted by the software Feature Extraction V10.5 (Agilent Technologies, Palo Alto, CA, USA).

Data preprocessing and statistical analysis were performed by LIMMA (LInear Model of Microarray Analysis) package [19]. The quality control of raw data was carried out according to MAQC (MicroArray Quality Control) project guidelines [20]. The intensity raw data were background subtracted by *normexp* method and normalized within-arrays by *LOESS* and between-arrays by *scale* methods.

Bayesian moderated t-statistic [21] was used to perform the statistical analysis and only genes with Benjamini and Hochberg [22] adjusted-p-value <0.05 were considered as differentially expressed.

*GeneCards* (http://www.genecards.org) [23], *Onto-Express* (http://vortex.cs.wayne.edu/ontoexpress/) [24], [25], and *COREMINE* (http://www.coremine.com/medical/) bioinformatics tools were adopted to build interaction networks among the differentially expressed genes and to perform an accurate screening of related scientific evidence.

### Microarray data validation by RT-qPCR

The same RNA samples used in microarray experiments were used to perform RT-qPCR experiments. Total RNAs were reverse transcribed with random and oligo-dT primers by the QuantiTect Reverse Transcription kit (Qiagen, Valencia, CA, USA). PCR primers were designed by the Beacon Designer 4.0 software (Premier Biosoft International, Palo Alto, CA, USA) and synthesized by Sigma-Aldrich (Sigma-Aldrich, St.Louis, MO, USA). The primer sequences are listed in table 1.

**Table 1:** Housekeeping genes, target genes and RT-qPCR primers.

Housekeeping genes			
Gene Symbol	RefSeq mRNA	Forward Primer	Reverse Primer
Mapk6	NM_031622.2	5′GCCACACAAACCGCTGAC 3′	5′CCGTTGGGAAAGAGTAGATGC3′
Kdm2b	NM_001100679	5′GCAAGCAAGTCACCAAGG 3′	5′TCGTTTCAGATTCCAAAGGG 3′
Psmd4	NM_031331	5′AGATGATGCCCTACTGAAGATGAC 3′	5′GACGCTCTGAAGGAACTCTGG3′
Cypa	NM_017101	5′ CAAGACTGAGTGGCTGGATGG 3′	5′GCTACAGAAGGAATGGTTTGATGG3′
B2mg	NM_012512	5′TCAAGTGTACTCTCGCCATCC 3′	5′GCAAGCATATACATCGGTCTCG3′
Bact	NM_31144	5′CCACACCCGCCACCAGTTC3′	5′GACCCATACCCACCATCACACC 3′

Subcutaneous adipose tissue			
Gene Symbol	RefSeq mRNA	Forward Primer	Reverse Primer
Scarb1	NM_031541	5′GCAGTGATGATGGAGGACAAGCC 3′	5′GAAGACGGTGAAGACGCCAGAAC 3′
Igfbp2	NM_013122	5′CCTCTGGAACATCTCTACTC 3′	5′ACTGGCTGTGGTTTACTG 3′
Ascl5	NM_053607	5′TGGGATGCTGGGTGTTTG 3′	5′TACTTCCTGTGTCTTCTCTGG 3′
Apod	NM_012777	5′AGTTGAGGGTGAAGCCAAACAGAG 3′	5′GCAGGAATACACGAGGGCATAGC 3′
Cebpb	NM_024125	5′ATCGACTTCAGCCCCTACCT3′	5′GGCTCACGTAACCGTAGTCG3′

Liver			
Gene Symbol	RefSeq mRNA	Forward Primer	Reverse Primer
Gk	NM_024381	5′GAATCCCACTCAGCCATTTGC 3′	5′TTCAAGACTCCAGACACCAACC3′
Me1	NM_012600	5′GGCACAGGCGAGTGAGAGG 3′	5′TTATCCGAAGAGCAGCAAGAAGG3′
Thrsp	NM_012703	5′GGCAGTGAGGCTGAGAATGAG 3′	5′GATGTGATGGAGGCTGGAGAAG 3′
Ldlrap1	NM_001109271	5′ACAGTCACGCAGGGAGAGG 3′	5′CAGAGAAGAGGAACAAGCAACAC3′
Insig2	AY156086	5′CCCCTAGACGGATGTGTTGAAG 3′	5′AAGGTGAACTGTGGACTGAAGC 3′
Dbp	NM_012543	5′ACAAGAACAATGAAGCAGCCAAG 3′	5′CAGGTGTAAGTCTGAACAAGGAAC3′
Tef	NM_019194	5′GCATCACCACCATCTTCCTCTAC3′	5′AGTTCACATCAACCTCCACACAG3′

RT-qPCR was performed by the iCycler iQ instrument (Biorad, Hercules, CA, USA) using the iQ SYBR Green Supermix (Biorad, Hercules, CA, USA). The amplification protocol was: 3 min at 95°C (DNA polymerase activation), then 40 cycles at 95°C for 30 sec (denaturation step), 58–62°C (depending on primer Tm) for 60 sec (annealing step) and 72°C for 30 sec (extension step). Afterwards, a gradual increase in temperature from 55°C to 95°C at 0.5°C/10 sec was utilized to build a melting curve. For each primer pair, amplification efficiency was tested using five serial dilutions of cDNA carried out in duplicate. To reduce the effects of the biological variation on amplification efficiency, a cDNA sample obtained by pooling the RNAs from all the eight control samples was used. For all primer pairs amplification efficiency was between 90 and 110% and R^2^ was>0.99. The stability of six housekeeping genes (Mapk6, Kdm2b, Psmd4, Cypa, B2mg, Bact) was evaluated by using geNorm software [26], see table 1. geNorm identified three housekeeping genes as stable which were used to normalize the expression values of the target genes: Psmd4, Cypa and B2mg genes in the adipose tissue and Bact, Kdm2b and B2mg genes in the liver.

Each sample was run in triplicate to calculate the standard deviation (SD) for the three experimental replicates. Only experiments with SD <0.4 for each group of replicates were considered. The relative expression levels for the target genes in T_1_AM treated respect to T_1_AM untreated tissues were calculated by geNorm method and reported as fold increase or decrease.

One- and two-tailed Wilcoxon signed rank tests in the Mann-Whitney version were applied to evaluate the statistical significance of RT-qPCR results by using a threshold p-value <0.05.

## Results

### Tissue T_1_AM concentration

Chronic T_1_AM administration did not produce any apparent effect on animal behaviour. In the control group, T1AM concentration averaged 5.38±1.30 pmol/g in liver and 0.36±0.07 pmol/g in adipose tissue. After 5 days of administration of exogenous T_1_AM (10 mg/Kg twice a day) liver concentration increased to 288.04±22.91 pmol/g and adipose tissue concentration increased to 8.35±2.45 pmol/g (P<0.01 in both cases).

Although tissue processing was not optimized for T3 and T4 assay, these substances were also identified in liver samples by HPLC/MS-MS [8], and no significant difference was observed in treated animals, although there was a trend for decreased T3 (15.92±2.14 vs 17.98±1.13 pmol/g) and increased T4 (194.14±13.24 vs 155.68±14.22 pmol/g).

### Microarray results

T_1_AM chronic administration impacted the expression of a greater number of genes in rat subcutaneous adipose tissue than in liver. Specifically, 378 genes were differentially expressed in adipose tissue, 268 up-regulated and 110 down-regulated, while in liver the differentially expressed genes were 114, 63 up-regulated and 51 down-regulated (see Table S1 and S2). Complete information about the microarray experiments and results can be retrieved from the ArrayExpress database at the European Bioinformatics Institute (EBI) (http://www.ebi.ac.uk/arrayexpress/) using the following accession numbers: E-MTAB-2177 for the subcutaneous adipose tissue and E-MTAB-2178 for the liver.

To identify groups of genes involved in cellular processes that might explain the observed T_1_AM phenotypical effects, the two lists of differentially expressed genes were investigated by using *Onto-Express*, *GeneCards* and *COREMINE* bioinformatics tools and by an accurate screening of the scientific literature. In the subcutaneous adipose tissue, 19 genes involved in lipoprotein functions, lipolysis and beta-oxidation and adipogenesis were identified (table 2), while in liver seven genes involved in lipid metabolism (table 3) appeared as the most relevant.

**Table 2:** Differentially expressed genes in the subcutaneous adipose tissue annotated by *Onto-Express*, *GeneCards* and *COREMINE*.

	Genes	Fold-Change direction
*Lipoprotein functions*	Ldlrap1 (low density lipoprotein receptor adaptor protein 1)	↑
	Lrp10 (low-density lipoprotein receptor-related protein10)	↑
	Apod (Apolipoprotein D)	↑
	Scarb1 (scavenger receptor class B, member 1)	↑
	Sirt6 (sirtuin 6)	↑
	Osbpl5 (oxysterol binding protein-like 5)	↑
*Lipolysis and Beta-oxidation*	Adra2c (adrenergic, alpha-2C-, receptor)	↓
	G0s2 (G(0)/G(1) switch gene 2)	↓
	Acsl5 (acyl-CoA synthetase long-chain family member 5)	↑
	Pex5 (peroxisomal biogenesis factor 5)	↑
*Adipogenesis*	Stat5b (signal transducer and activator of transcription 5B)	↑
	Cebpb (CCAAT/enhancer binding protein (C/EBP), beta)	↓
	Pmp22 (peripheral myelin protein 22)	↑
	Sirt2 (sirtuin 2)	↑
	Nolc1 (nucleolar and coiled-body phosphoprotein 1)	↓
	Igfbp2 (insulin-like growth factor binding protein 2)	↓
	Dmpk (dystrophia myotonica-protein kinase)	↑
	Paqr3 (progestin and adipoQ receptor family member III)	↑
	Pla2g2a (phospholipase A2, group IIA (platelets, synovial fluid)	↓

**Table 3:** Differentially expressed genes in liver annotated by *Onto-Express*, *GeneCards* and *COREMINE*.

	Genes	Fold-Change direction
*Lipid metabolism*	Ldlrap1 (low density lipoprotein receptor adaptor protein 1)	↑
	Gk (glycerol kinase)	↓
	Me1 (malic enzyme 1, NADP(+)-dependent, cytosolic)	↓
	Insig2 (insulin induced gene 2)	↓
	Thrsp (thyroid hormone responsive)	↑
	Dbp (D site of albumin promoter (albumin D-box) binding protein)	↑
	Tef (thyrotrophic embryonic factor)	↑

### RT-qPCR results

To validate the microarray results, five differentially expressed genes were selected for the subcutaneous adipose tissue: Scarb1, Acsl5, Apod, Igfbp2, and Cebpb and seven for the liver: Gk, Me1, Thrsp, Ldlrap 1, Insig2, Dbp and Tef.

All these genes were chosen based on their p-values and their biological relevance in explaining the T1AM molecular mechanisms of action.

In the adipose tissue the differential expression was confirmed for Igfbp2, Acsl5, Scarb1, Apod and Cebpb, although Cebpb did not reach the statistical significance

**Figure 2:**
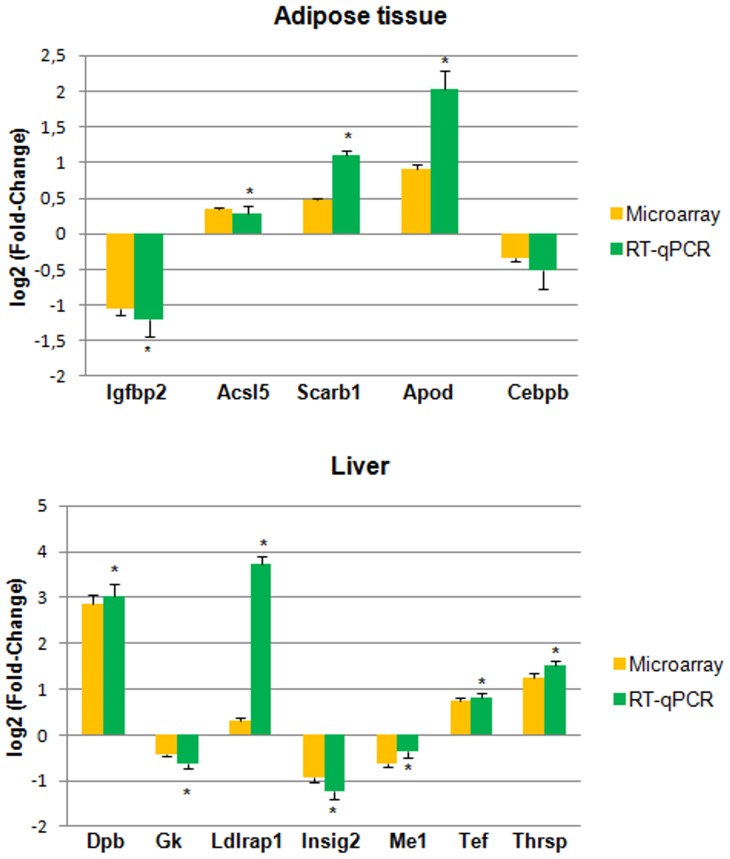
Changes in gene expression evidenced by microarrays were confirmed by RT-qPCR for four of the five genes tested in the subcutaneus adipose tissue (the differential expression of Cebpb was not statistically significant) and for all the seven genes tested in the liver. Data reported as: log2 fold-change ± SE; *: p≤0.05.

In liver the differential expression was confirmed for all the tested genes (figure 2).

## Discussion

T_1_AM is regarded as a novel chemical messenger able to produce a variety of functional effects, but the underlying molecular mechanisms have not been fully understood. In particular, it is still unknown whether T_1_AM can modulate gene expression, although this seems a likely possibility, since some effects are long lasting, and this applies in particular to modulation of fatty acid metabolism [6].

In order to investigate the molecular mechanisms underlying the effects of T_1_AM, gene expression profiles were analyzed in the subcutaneous adipose tissue and in liver of eight rats chronically treated with T_1_AM as compared with eight untreated rats. These tissues were selected because of their central role in the regulation of energy homeostasis and lipid/carbohydrate metabolism [27], [28].

Prolonged T_1_AM administration affected the expression of many more genes in rat subcutaneous adipose tissue than in liver, suggesting a greater responsiveness to T_1_AM of adipose tissue compared to liver. Notably, the assay of tissue T_1_AM showed that in our experimental model endogenous concentration increased by about one order of magnitude (20-fold in adipose tissue and 50-fold in liver), without significant changes in tissue thyroid hormone concentration. Therefore, the effects that we observed might have physiological or pathophysiological importance.

Our investigation cannot provide direct mechanistic information on lipid metabolism modulation by T_1_AM, since indices of lipid metabolism were not determined. In addition, dose-response studies were not performed, and only a single dose was administered for a relatively short period of time. However, some of the observed changes in gene expression are consistent with the responses that have been reported in previous investigations, and deserve a specific discussion of their potential functional implications. We also compare the transcriptional response to T_1_AM with the known genomic effects of thyroid hormone.

### Effects of T_1_AM on subcutaneous adipose tissue

Among the 378 genes differentially expressed in the subcutaneous adipose tissue, 19 genes appeared to have a significant role in molecular mechanisms relevant to metabolic effects (table 2).

#### T_1_AM up-regulates genes related to lipoprotein function

Lipoproteins are delegated to transport lipids, which are insoluble in blood, in the circulatory system. Most of adipocyte cholesterol originates from circulating lipoproteins [29]. *De novo* synthesis of cholesterol is, in fact, low in the adipose tissue, as observed in early studies, which reported that the rate of cholesterol synthesis in fat cells is only 4% of that of liver [30].

Some genes regulated by T_1_AM are related to lipoprotein function and five of them are of particular interest: **Ldlrap1** (low density lipoprotein receptor adaptor protein 1), **Lrp10** (low-density lipoprotein receptor-related protein10), **Apod** (Apolipoprotein D), **Scarb1** (scavenger receptor class B, member 1) and **Sirt6** (sirtuin [silent mating type information regulation 2 homolog] 6).

The **Ldlrap1** product is an adaptor protein required for efficient endocytosis of low density lipoprotein receptor (LDLR), which plays a crucial role for the removal of circulating LDLs (Low Density Lipoproteins) [31]. The protein encoded by this gene stabilizes the association between LDLR and LDL and promotes the internalization of the LDL-LDLR complex [31]. Alterations in the bond between LDL and LDLR impede the endocytosis of the complex and lead to accumulation of LDLs in plasma.

**Lrp10** belongs to the LDLR family and its product mediates the cellular uptake of cholesterol-rich VLDLs (Very Low Density Lipoproteins) remnants *in vitro* [32]. Sugiyama and colleagues demonstrated that Lrp10, through the interaction with apoE that is abundant in the VLDL remnants, is involved in their blood clearance [32]. Lrp10 is also a molecular target of Ginko Biloba that is known to have cholesterol-lowering effect [33].

**Apod** is an apolipoprotein structurally similar to the lipocalin family proteins that is responsible for lipid transport. Reduced Apod expression alters lipid metabolism [34]. Plasma Apod is a component of HDLs (High Density Lipoproteins) involved in the “reverse cholesterol transport” by which the cholesterol is transferred from peripheral tissues to the liver for biliary excretion [35]. Apod modulates the activity of lecithin: cholesterol acyltransferase (LCAT), a HDL-bound enzyme that catalyzes the conversion of free cholesterol to CE that is then recruited into the HDL core. Increased cholesterol esterification by LCAT is observed in presence of Apod and formation of Apod-LCAT complex has a stabilizing effect on LCAT [36]. By enhancing cholesterol esterification through LCAT, Apod indirectly promotes reverse cholesterol transport [37]. Moreover, a covalent cross-link between Apod and Apoa-II, a structural component of HDL, has been identified [38].

The **Scarb1** gene codifies an HDL transmembrane receptor that mediates CE transfer from plasma HDL to tissues without HDL particle degradation (CE selective up-take) [39]. HDL-Scarb1 interaction induces the formation of a hydrophobic channel by which the HDL unloades the CE. Cholesterol-depleted HDL dissociates from the receptor and re-enters the circulation to capture other molecules of peripheral cholesterol [40].

Since Scarb1 regulates HDL cholesterol levels, its decrease has been associated with increased susceptibility to atherosclerosis: Scarb1 KO mice showed elevated HDL cholesterol and reduced selective HDL cholesterol clearance [41], [42]. In addition, the disruption of Scarb1 gene in atherosclerotic mice (APOE ^-^/^-^) accelerates the onset of atherosclerosis [43].

**Sirt6** codifies a member of sirtuin family that has NAD-dependent deacetylase and ADP-ribosyltransferase activities [44]–[46]. It has been recently observed that transgenic mice overexpressing Sirt6 and fed with high fat diet accumulate significantly less LDL-cholesterol compared with their wild–type littermates [47].

To summarize, T_1_AM, by modulating the expression of genes related to lipoprotein function, potentially affects cholesterol homeostasis. This hypothesis is corroborated by the upregulation of another gene, **Osbpl5** (oxysterol binding protein-like 5), which codifies a member of the oxysterol-binding protein (OSBP) family that controls oxysterol activity [48]. Oxysterols, oxygenated derivatives of cholesterol, are particularly potent inhibitors of cholesterol biosynthesis [49]. Therefore, investigations specifically targeted to evaluate the effect of chronic T1AM administration on cholesterol homeostasis may be appropriate.

#### T_1_AM regulates genes related to lipolysis and beta-oxidation

Lipolysis hydrolyzes triglycerides and releases glycerol and free fatty acids. Some genes related to lipolysis, like **Adra2c** (adrenergic, alpha-2C-, receptor) and **G0s2** ((G(0)/G(1) switch gene 2)) are down-regulated by T_1_AM.

**Adra2c** is a target of catecholamines that are important regulators of fat cell lipolysis [50]. Sustained lipid mobilization and an increase in energy expenditure were observed during administration of an alpha2-adrenoceptor antagonist in dogs and humans [51], [52].

The **G0s2** protein negatively regulates the activity of the adipose triglyceride lipase (ATGL), which catalyzes the first step in the hydrolysis of triglycerides. G0s2 protein binds directly to ATGL and reduces ATGL-mediated lipolysis by inhibiting its hydrolase activity [53]. In Hela cells, G0S2 over-expression prevents the ATGL-mediated lipid droplet degradation as well as basal and stimulates lipolysis in cultured adipocytes, whereas down-regulation of endogenous G0S2 enhances adipocyte lipolysis [53].

T_1_AM up-regulates the expression of genes linked to beta-oxidation, particularly **Acsl5** (acyl-CoA synthetase long-chain family member 5) and **Pex5** (peroxisomal biogenesis factor 5).

In the cytoplasm, free fatty acids are converted into acyl-CoA thioesters by acyl-CoA synthetases (ACSs). Then, they are directed toward *de novo* lipid synthesis to store energy or toward beta-oxidation both in mitochondria and in peroxisomes to produce ATP [54]. Long-chain ACSs (ACSLs) act on fatty acids containing 12–22 carbons [55]. **Acsl5** is the only ACSL isoform known to be located on the mitochondrial outer membrane and it probably plays an important role in the beta-oxidation of fatty acids [56]. In support of this hypothesis an increase of Acsl5 protein and mRNA after food deprivation has been observed [57]. Moreover, Acsl1 and Acsl4, but not Acsl5, are inhibited by Triascin C [58] that blocks the *de novo* triglyceride synthesis [59], suggesting that Acsl5 is not involved in triglyceride synthesis.

The **Pex5** gene codes a protein involved in the biogenesis of peroxisomes, which are organelles where very long chain fatty acids undergo the initial steps of beta-oxidation [60], [61]. These data suggest that T_1_AM promotes both triglyceride lipolysis and beta-oxidation, according to increased lipid utilisation, which has been observed in different experimental models [6], [12].

#### T_1_AM regulates the expression of genes related to adipogenesis

The amount of body fat depends on the size and number of adipocytes. Besides mature adipocytes, adipose tissue contains multipotent mesenchymal cells and pre-adipocytes able to proliferate after specific stimuli [62]. If food intake exceeds energy consumption, mature adipocytes undergo hypertrophy (increase in size) and hyperplasia (increase in number) [62]. The latter, also known as adipogenesis, is based on recruitment, proliferation and differentiation of pre-adipocytes [63].

Several transcription factors, including members of the C/EBP family, are induced during adipocyte differentiation and play an important role in the regulation of adipocyte gene expression [64]. **Cebpb**, down-regulated by T_1_AM, is the first player in adipogenesis, being responsible of C/EBPalfa and PPARgamma activation [64], [65].

Adipocyte gene expression is also affected by Signal Transducers and Activators of Transcription (STATs) [66]. **Stat5b**, up-regulated by T_1_AM, is activated in the early phase of the differentiation process and is a positive regulator of proliferation [67]. However, a continuous and excessive activation of Stat5b becomes inhibitory for adipogenesis [65].

Other genes regulated by T_1_AM, including **Pmp22** (peripheral myelin protein 22), **Sirt2** (sirtuin [silent mating type information regulation 2 homolog] 2), **Nolc1** (nucleolar and coiled-body phosphoprotein 1) and **Igfbp2** (insulin-like growth factor binding protein 2, 36kDa) are implicated in adipogenesis.

**Pmp22**, up-regulated by T_1_AM, belongs to the Growth Arrest Specific (GAS) gene family. The genes of this family regulate cellular growth by blocking mitotic division in response to extracellular signals [68]. In mice 3T3-L1, during pre-adipocyte maturation, GAS genes are up-regulated and Pmp22 gene exerts an inhibitory effect on adipogenesis [69].

**Sirt2**, up-regulated by T_1_AM, codes for a member of the sirtuin family. In mouse 3T3-L1 pre-adipocytes, Sirt2 overexpression inhibits adipocyte differentiation [70], while Sirt2 downregulation promotes adipogenesis [70]. Sirt2 suppresses adipogenesis by deacetylating FOXO1, which ties PPARgamma and represses its transcriptional activity [70].

**Nolc1**, down-regulated by T_1_AM, codes for a member of the retinoblastoma family. These proteins are phosphorylated by cyclins to promote cell proliferation in a variety of cells [71]. In adipose tissue, cell proliferation is stimulated by FGF10 through activation of the Ras/Map pathway followed by cyclin D2-dependent NOLC1-phosphorylation [72].

**Igfbp2**, down-regulated by T_1_AM, codifies a member of the IGF binding protein family that sequesters the IGFs in the extracellular environment and limits their access to the signalling receptors [73]. In particular, Igfbp2 inhibits IGF1-IGF1R interaction by sequestering IGF1 [73] that is an inducer of pre-adipocyte differentiation [74]. Whether Igfbp2 exerts an inhibitory effect on pre-adipocyte differentiation by sequestering IGF1 is unknown [73], but it has been recently observed that mice overexpressing Igfbp2 show increased fat mass [75]. These data raise the hypothesis that T_1_AM controls adipose tissue expansion by inhibiting adipogenesis.

On the other hand, up-regulation of **Dmpk** (dystrophia myotonica-protein kinase) gene might reduce adipocyte hypertrophy. This gene encodes a serine/threonine protein kinase, whose deficiency appears to be a risk factor for adiposity. Dmpk KO mice fed with high-fat diet exhibit increased body weight and fat mass, due to adipocyte hypertrophy [76].

Finally, given that the adipose tissue expansion requires the formation of new vessels, [77]–[79] the regulation of angiogenesis-related genes, like **Paqr3** (progestin and adipoQ receptor family member III) and **Pla2g2a** (phospholipase A2, group IIA platelets, synovial fluid) might represent an additional molecular mechanism by which T_1_AM inhibits adipogenesis.

**Paqr3** gene, up-regulated by T_1_AM, codifies an adiponectin receptor [80] that has been reported to inhibit angiogenesis by suppressing VEGF signalling [81].

**Pla2g2a** gene, down-regulated by T_1_AM, codifies a phospholipase that catalyzes the sn-2 acyl- hydrolysis of phospholipids. Pla2g2a inhibition has been shown to reduce the formation of capillary-like tubes [82].

### Effects of T_1_AM on liver

Although 114 genes were differentially expressed, no significant effect was observed on the transcription of toxicity genes, consistent with the observed high tolerability of T_1_AM administration. However, this conclusion needs confirmation by further investigations, since our investigation covered a limited time span. In addition, since a large number of genes was affected, specific analysis is required to exclude toxic effects arising from interaction between gene products Gene expression analysis, however, highlighted a potential impact of T_1_AM on lipid metabolism in liver, as seven genes linked to lipid metabolism were identified: **Ldlrap 1** (low density lipoprotein receptor adaptor protein 1), **Insig2** (insulin induced gene 2), **Thrsp** (thyroid hormone responsive), **Gk** (glycerol kinase), **Me1** (malic enzyme 1, NADP(+)-dependent, cytosolic), **Dbp** (D site of albumin promoter (albumin D-box) binding protein) and **Tef** (thyrotrophic embryonic factor).

**Ldlrap 1**, up-regulated by T_1_AM both in liver and adipose tissue, is required for efficient LDL/LDL receptor endocytosis in hepatocytes [83]. **Insig2**, down-regulated, is able to reduce lipogenesis by inhibiting sterol regulatory element-binding proteins (SREBPs) [84]. **Thrsp**, up-regulated by T_1_AM and also by thyroid hormones, is a positive regulator of lipogenesis [85]. The differential expression of Ldlrap1, Insig2 and Thrsp suggests increased lipid internalization and storage due to T_1_AM administration. On the other hand, **Gk** and **Me1**, key enzymes in the biosynthesis of triglycerides and fatty acids [86]–[88], were both downregulated by T_1_AM.

Finally, in liver, T_1_AM seems to impact the circadian rhythm of lipid metabolism, positively regulating the expression of two output mediators of the circadian clock, **Dbp** and **Tef**. Both proteins, members of the PAR (Proline and Acidic amino acid Rich) subfamily of transcription factors, contribute to the circadian transcription of genes encoding acyl-CoA thioesterases, leading to a cyclic release of fatty acids from thioesters. In turn, fatty acids act as ligands for PPARα (Peroxisome Proliferator-Activated Receptors α), which stimulates the transcription of genes encoding proteins involved in the uptake and/or metabolism of lipids and cholesterol [89].

Anyhow, further investigation is required to better elucidate the T1AM effect on lipid metabolism in liver.

### Transcriptional effects of T_1_AM versus thyroid hormone

Stimulation of lipid catabolism has also been considered as an effect of thyroid hormone [90]. A comparison between the transcriptional response to T_1_AM and the genomic effects of T_3_ and other hormones, as derived from literature data, is shown in Table 4. For many crucial T_1_AM targets no significant effect has been reported for thyroid hormone. Even more interesting, Cebpb, GK and Me1 transcription was inhibited by T_1_AM, while the opposite effect has been reported both for T_3_ and for insulin. Therefore, T_1_AM appears to determine significant genomic effects, which are not reproduced by thyroid hormone. Since it is likely, although not formally demonstrated yet, that T_1_AM is synthesized from T_3_, it would be interesting to investigate if some of the metabolic effects usually attributed to the latter may actually be mediated by T_1_AM.

**Table 4:** Comparison of the transcriptional response to T_1_AM with the known genomic effects of thyroid hormone, Insulin and Cortisol (up-regulated  = ↑, down-regulated  = ↓, not regulated or no data available  =  -).

Genes	T_1_AM	Thyroid hormone	Insulin	Cortisol
Ldlrap1	↑	-	-	-
Lrp10	↑	-	-	-
Apod	↑	-	-	↑[91]
Scarb1	↑	-	-	-
Sirt6	↑	-	-	-
Osbpl5	↑	-	-	-
Adra2c	↓	-	-	-
G0s2	↓	-	↑[92]	-
Acsl5	↑	-	-	-
Pex5	↑	-	-	-
Stat5b	↑	-	-	-
Cebpb	↓	↑ [93]	↑ [94]	↑ [91]
Pmp22	↑	-	-	-
Sirt2	↑	-	-	-
Nolc1	↓	-	-	-
Igfbp2	↓	-	-	↑[95]
Dmpk	↑	-	-	-
Paqr3	↑	-	-	-
Pla2g2a	↓	↓ [96]	-	-
Gk	↓	↑[97]	↑[97], [98]	-
Me1	↓	↑ [99]	↑[99]	-
Thrsp	↑	↑[100]	↑[101]	-
Insig2	↑	-	↑[102]	-
Dbp	↑	-	-	-
Tef	↑	-	-	-

## Conclusions

*In vivo* T_1_AM administration produces significant transcriptional effects, more evident in adipose tissue than in liver that might contribute to explain the increase of lipid metabolism and the reduction of fat mass. Therefore we suggest that transcriptional effects might provide the basis for the reported effectiveness of T_1_AM as a lipolytic agent and gain importance in view of a possible clinical use of T_1_AM, since no effect on the expression of genes linked to toxicity has been observed so far.

Furthermore, T_1_AM influences the expression of several genes relating to lipoprotein metabolism suggesting that T_1_AM-mediated mechanisms may also play an important role in the regulation of cholesterol homeostasis.

To our knowledge this is the first study of gene expression in response to T_1_AM administration. Further investigation by *in vitro* and *in vivo* functional studies is required to confirm our observations and to understand its molecular mechanism. T_1_AM is known to interact with high affinity with TAAR1, a G protein-coupled receptor, but the putative transduction pathway leading to modulation of gene expression is unknown. While T_1_AM does not activate nuclear thyroid hormone receptors [7] it has not been excluded that additional targets may exist.

## Supporting Information

Table S1 **Adipose tissue Uni-mRNA and GO results.**
(XLS)

Table S2 **Liver tissue Uni-mRNA and GO results.**
(XLS)
